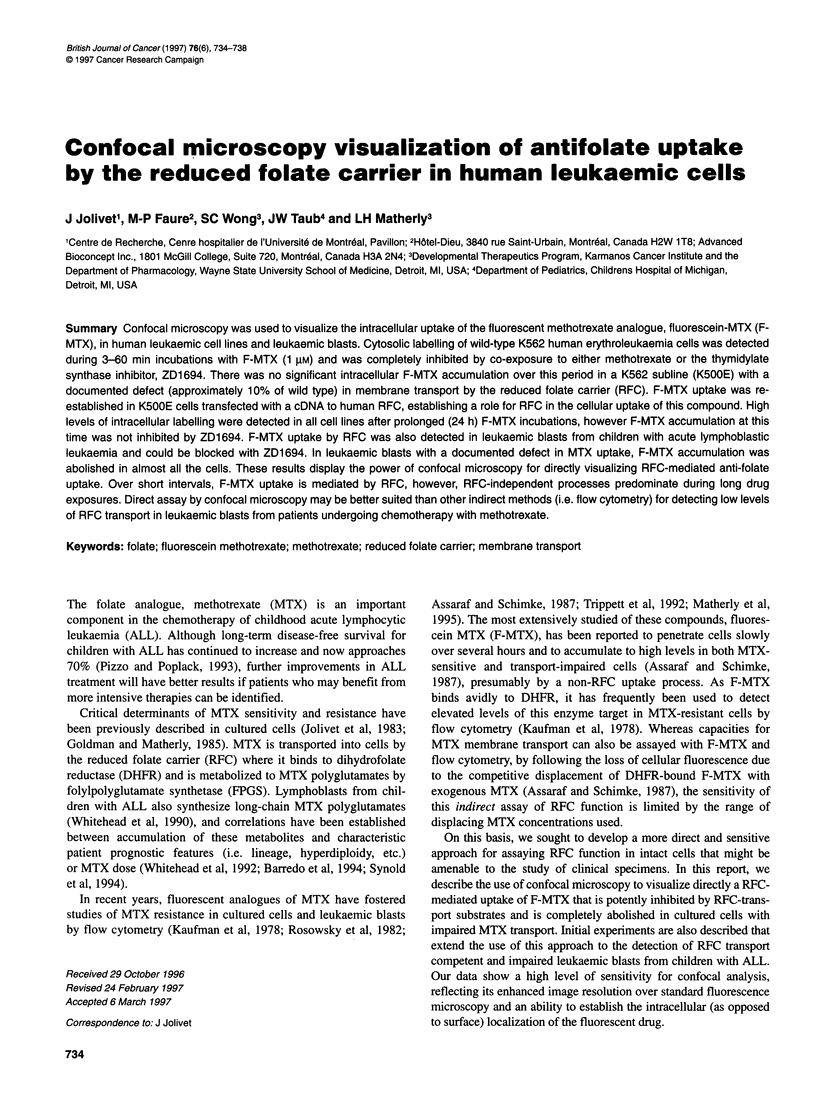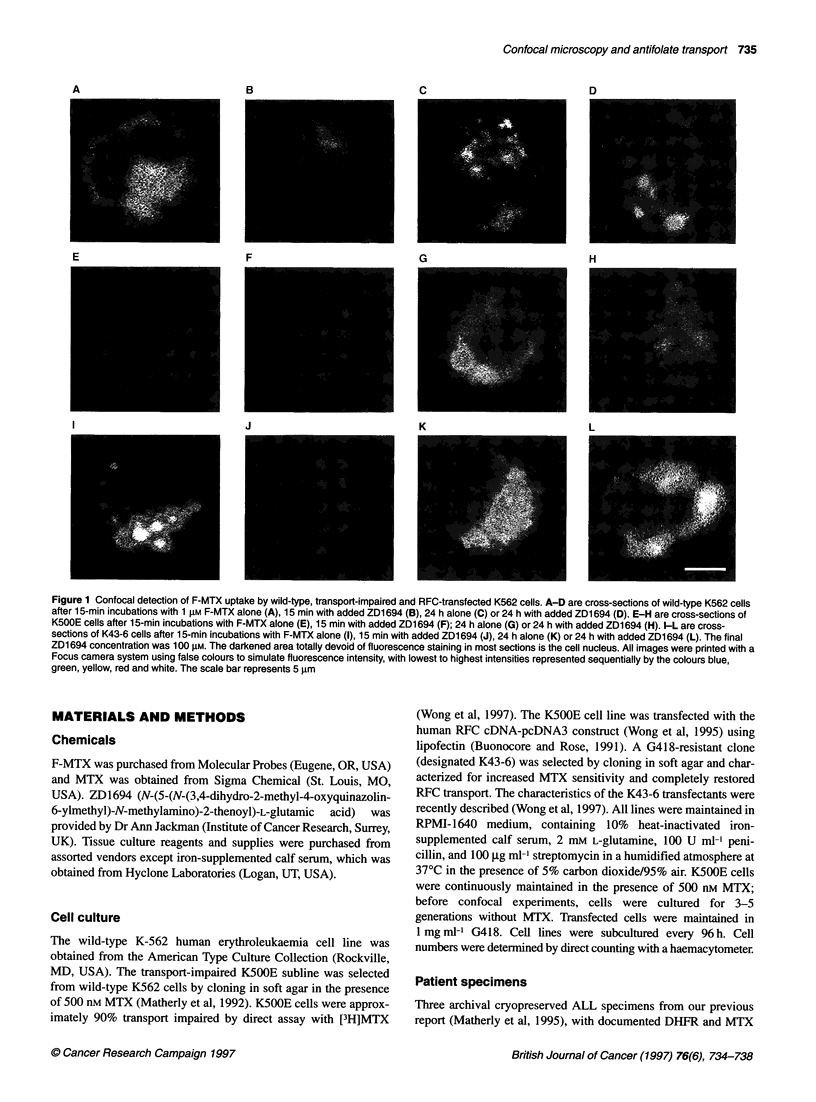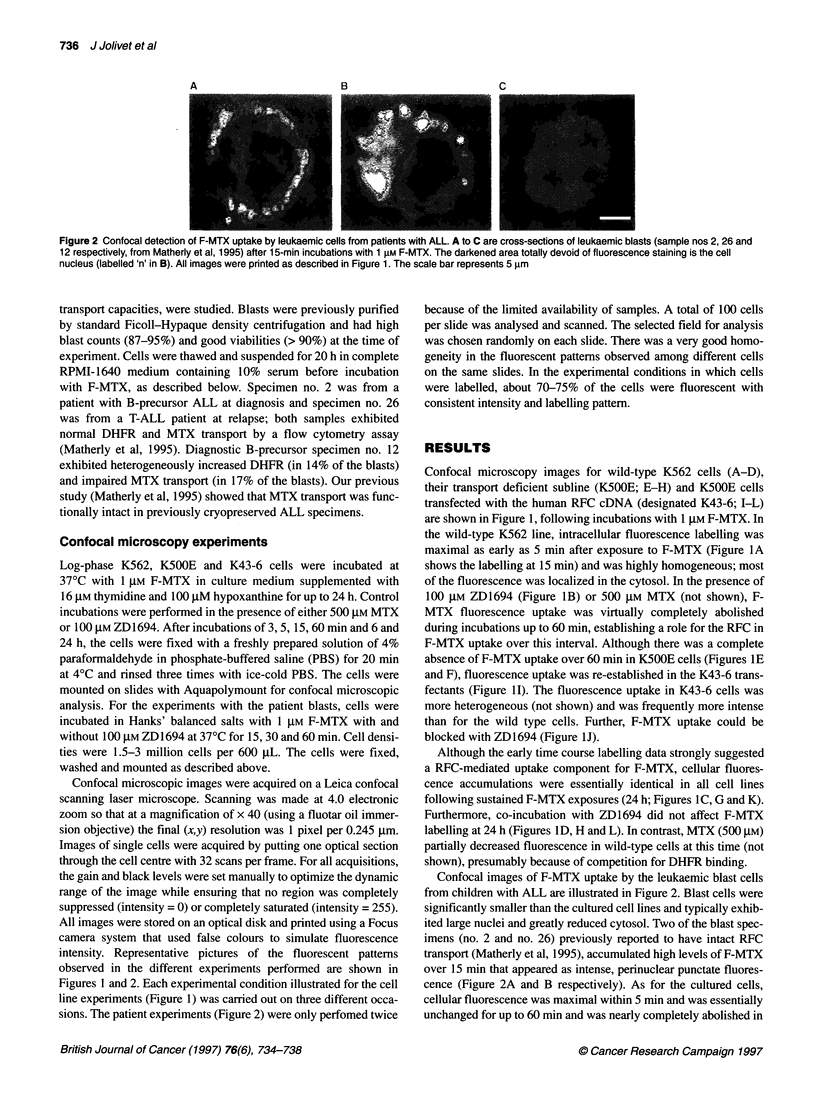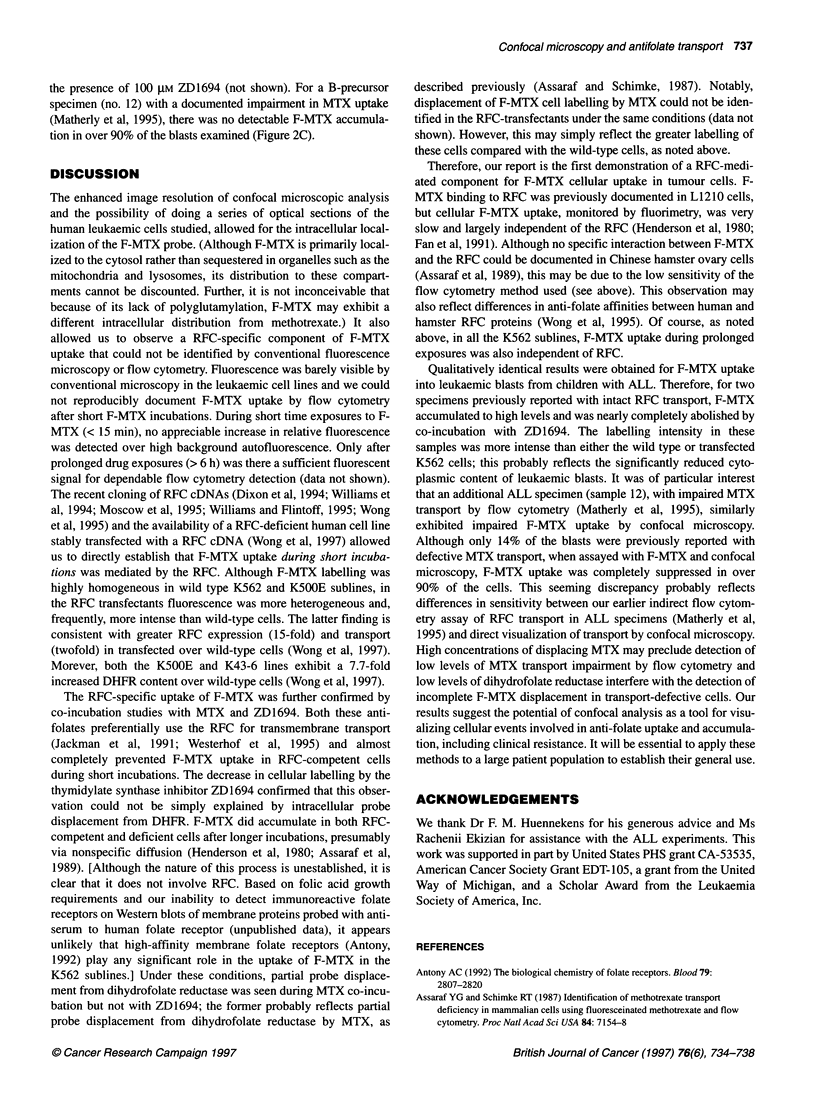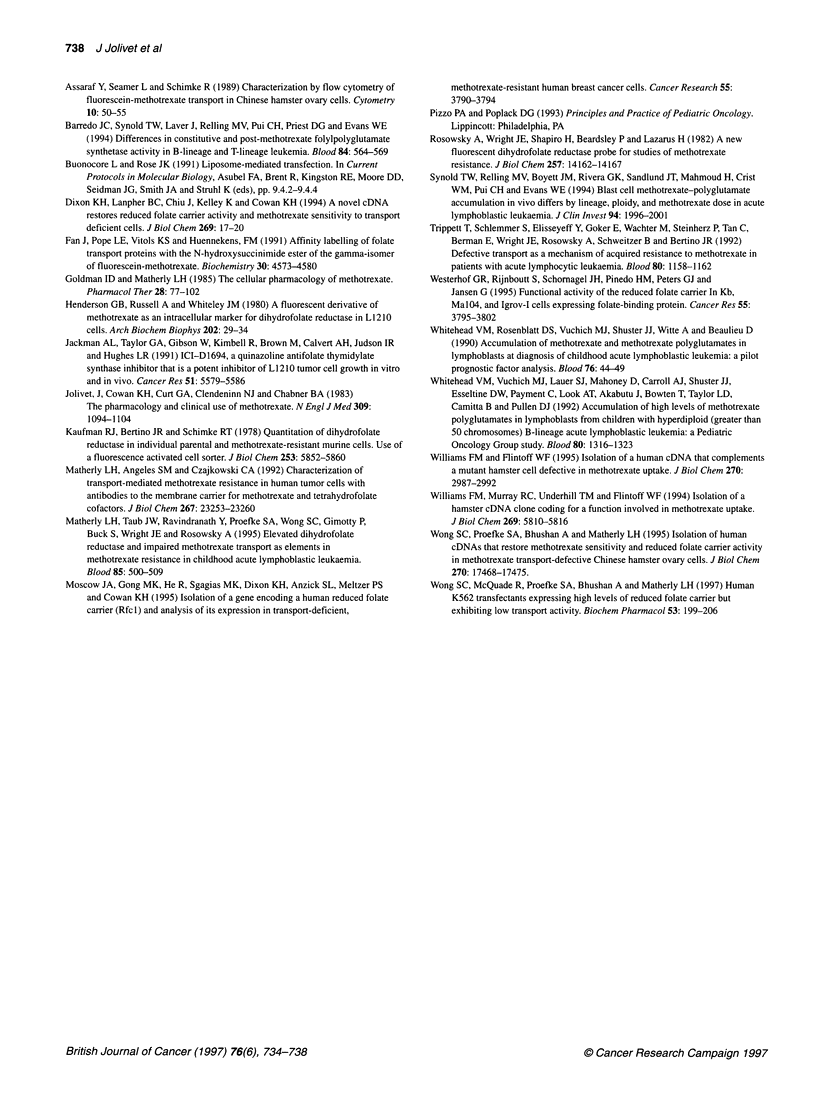# Confocal microscopy visualization of antifolate uptake by the reduced folate carrier in human leukaemic cells.

**DOI:** 10.1038/bjc.1997.454

**Published:** 1997

**Authors:** J. Jolivet, M. P. Faure, S. C. Wong, J. W. Taub, L. H. Matherly

**Affiliations:** Centre de Recherche, Centre hospitalier de l'UniversitÃ© de MontrÃ©al, Pavillon, Canada.

## Abstract

**Images:**


British Journal of Cancer (1997) 76(6), 734–738
© 1997 Cancer Research Campaign

### Confocal microscopy visualization of antifolate uptake by the reduced folate carrier in human leukaemic cells

**J Jolivet[1], M-P Faure[2], SC Wong[3], JW Taub[4] and LH Matherly[3]**

[1]Centre de Recherche, Cenre hospitalier de l'Université de Montréal, Pavillon; [2]Hôtel-Dieu, 3840 rue Saint-Urbain, Montréal, Canada H2W 1T8; Advanced Bioconcept Inc., 1801 McGill College, Suite 720, Montréal, Canada H3A 2N4; [3]Developmental Therapeutics Program, Karmanos Cancer Institute and the Department of Pharmacology, Wayne State University School of Medicine, Detroit, MI, USA; [4]Department of Pediatrics, Childrens Hospital of Michigan, Detroit, MI, USA

**Summary** Confocal microscopy was used to visualize the intracellular uptake of the fluorescent methotrexate analogue, fluorescein-MTX (F-MTX), in human leukaemic cell lines and leukaemic blasts. Cytosolic labelling of wild-type K562 human erythroleukaemia cells was detected during 3–60 min incubations with F-MTX (1 μM) and was completely inhibited by co-exposure to either methotrexate or the thymidylate synthase inhibitor, ZD1694. There was no significant intracellular F-MTX accumulation over this period in a K562 subline (K500E) with a documented defect (approximately 10% of wild type) in membrane transport by the reduced folate carrier (RFC). F-MTX uptake was re-established in K500E cells transfected with a cDNA to human RFC, establishing a role for RFC in the cellular uptake of this compound. High levels of intracellular labelling were detected in all cell lines after prolonged (24 h) F-MTX incubations, however F-MTX accumulation at this time was not inhibited by ZD1694. F-MTX uptake by RFC was also detected in leukaemic blasts from children with acute lymphoblastic leukaemia and could be blocked with ZD1694. In leukaemic blasts with a documented defect in MTX uptake, F-MTX accumulation was abolished in almost all the cells. These results display the power of confocal microscopy for directly visualizing RFC-mediated anti-folate uptake. Over short intervals, F-MTX uptake is mediated by RFC, however, RFC-independent processes predominate during long drug exposures. Direct assay by confocal microscopy may be better suited than other indirect methods (i.e. flow cytometry) for detecting low levels of RFC transport in leukaemic blasts from patients undergoing chemotherapy with methotrexate.

**Keywords:** folate; fluorescein methotrexate; methotrexate; reduced folate carrier; membrane transport

The folate analogue, methotrexate (MTX) is an important component in the chemotherapy of childhood acute lymphocytic leukaemia (ALL). Although long-term disease-free survival for children with ALL has continued to increase and now approaches 70% (Pizzo and Poplack, 1993), further improvements in ALL treatment will have better results if patients who may benefit from more intensive therapies can be identified.

Critical determinants of MTX sensitivity and resistance have been previously described in cultured cells (Jolivet et al, 1983; Goldman and Matherly, 1985). MTX is transported into cells by the reduced folate carrier (RFC) where it binds to dihydrofolate reductase (DHFR) and is metabolized to MTX polyglutamates by folylpolyglutamate synthetase (FPGS). Lymphoblasts from children with ALL also synthesize long-chain MTX polyglutamates (Whitehead et al, 1990), and correlations have been established between accumulation of these metabolites and characteristic patient prognostic features (i.e. lineage, hyperdiploidy, etc.) or MTX dose (Whitehead et al, 1992; Barredo et al, 1994; Synold et al, 1994).

In recent years, fluorescent analogues of MTX have fostered studies of MTX resistance in cultured cells and leukaemic blasts by flow cytometry (Kaufman et al, 1978; Rosowsky et al, 1982; Assaraf and Schimke, 1987; Trippett et al, 1992; Matherly et al, 1995). The most extensively studied of these compounds, fluorescein MTX (F-MTX), has been reported to penetrate cells slowly over several hours and to accumulate to high levels in both MTX-sensitive and transport-impaired cells (Assaraf and Schimke, 1987), presumably by a non-RFC uptake process. As F-MTX binds avidly to DHFR, it has frequently been used to detect elevated levels of this enzyme target in MTX-resistant cells by flow cytometry (Kaufman et al, 1978). Whereas capacities for MTX membrane transport can also be assayed with F-MTX and flow cytometry, by following the loss of cellular fluorescence due to the competitive displacement of DHFR-bound F-MTX with exogenous MTX (Assaraf and Schimke, 1987), the sensitivity of this *indirect* assay of RFC function is limited by the range of displacing MTX concentrations used.

On this basis, we sought to develop a more direct and sensitive approach for assaying RFC function in intact cells that might be amenable to the study of clinical specimens. In this report, we describe the use of confocal microscopy to visualize directly a RFC-mediated uptake of F-MTX that is potently inhibited by RFC-transport substrates and is completely abolished in cultured cells with impaired MTX transport. Initial experiments are also described that extend the use of this approach to the detection of RFC transport competent and impaired leukaemic blasts from children with ALL. Our data show a high level of sensitivity for confocal analysis, reflecting its enhanced image resolution over standard fluorescence microscopy and an ability to establish the intracellular (as opposed to surface) localization of the fluorescent drug.

*Received 29 October 1996*
*Revised 24 February 1997*
*Accepted 6 March 1997*

*Correspondence to:* J Jolivet

**Figure 1** Confocal detection of F-MTX uptake by wild-type, transport-impaired and RFC-transfected K562 cells. **A–D** are cross-sections of wild-type K562 cells after 15-min incubations with 1 μM F-MTX alone (**A**), 15 min with added ZD1694 (**B**), 24 h alone (**C**) or 24 h with added ZD1694 (**D**). **E–H** are cross-sections of K500E cells after 15-min incubations with F-MTX alone (**E**), 15 min with added ZD1694 (**F**); 24 h alone (**G**) or 24 h with added ZD1694 (**H**). **I–L** are cross-sections of K43-6 cells after 15-min incubations with F-MTX alone (**I**), 15 min with added ZD1694 (**J**), 24 h alone (**K**) or 24 h with added ZD1694 (**L**). The final ZD1694 concentration was 100 μM. The darkened area totally devoid of fluorescence staining in most sections is the cell nucleus. All images were printed with a Focus camera system using false colours to simulate fluorescence intensity, with lowest to highest intensities represented sequentially by the colours blue, green, yellow, red and white. The scale bar represents 5 μm

### MATERIALS AND METHODS

### Chemicals

F-MTX was purchased from Molecular Probes (Eugene, OR, USA) and MTX was obtained from Sigma Chemical (St. Louis, MO, USA). ZD1694 (*N*-(5-(*N*-(3,4-dihydro-2-methyl-4-oxyquinazolin-6-ylmethyl)-*N*-methylamino)-2-thenoyl)-L-glutamic acid) was provided by Dr Ann Jackman (Institute of Cancer Research, Surrey, UK). Tissue culture reagents and supplies were purchased from assorted vendors except iron-supplemented calf serum, which was obtained from Hyclone Laboratories (Logan, UT, USA).

### Cell culture

The wild-type K-562 human erythroleukaemia cell line was obtained from the American Type Culture Collection (Rockville, MD, USA). The transport-impaired K500E subline was selected from wild-type K562 cells by cloning in soft agar in the presence of 500 nM MTX (Matherly et al, 1992). K500E cells were approximately 90% transport impaired by direct assay with [$^3$H]MTX (Wong et al, 1997). The K500E cell line was transfected with the human RFC cDNA-pcDNA3 construct (Wong et al, 1995) using lipofectin (Buonocore and Rose, 1991). A G418-resistant clone (designated K43-6) was selected by cloning in soft agar and characterized for increased MTX sensitivity and completely restored RFC transport. The characteristics of the K43-6 transfectants were recently described (Wong et al, 1997). All lines were maintained in RPMI-1640 medium, containing 10% heat-inactivated iron-supplemented calf serum, 2 mM L-glutamine, 100 U ml$^{-1}$ penicillin, and 100 μg ml$^{-1}$ streptomycin in a humidified atmosphere at 37°C in the presence of 5% carbon dioxide/95% air. K500E cells were continuously maintained in the presence of 500 nM MTX; before confocal experiments, cells were cultured for 3–5 generations without MTX. Transfected cells were maintained in 1 mg ml$^{-1}$ G418. Cell lines were subcultured every 96 h. Cell numbers were determined by direct counting with a haemacytometer.

### Patient specimens

Three archival cryopreserved ALL specimens from our previous report (Matherly et al, 1995), with documented DHFR and MTX

© Cancer Research Campaign 1997

**Figure 2** Confocal detection of F-MTX uptake by leukaemic cells from patients with ALL. **A** to **C** are cross-sections of leukaemic blasts (sample nos 2, 26 and 12 respectively, from Matherly et al, 1995) after 15-min incubations with 1 μM F-MTX. The darkened area totally devoid of fluorescence staining is the cell nucleus (labelled 'n' in **B**). All images were printed as described in Figure 1. The scale bar represents 5 μm transport capacities, were studied. Blasts were previously purified by standard Ficoll–Hypaque density centrifugation and had high blast counts (87–95%) and good viabilities (> 90%) at the time of experiment. Cells were thawed and suspended for 20 h in complete RPMI-1640 medium containing 10% serum before incubation with F-MTX, as described below. Specimen no. 2 was from a patient with B-precursor ALL at diagnosis and specimen no. 26 was from a T-ALL patient at relapse; both samples exhibited normal DHFR and MTX transport by a flow cytometry assay (Matherly et al, 1995). Diagnostic B-precursor specimen no. 12 exhibited heterogeneously increased DHFR (in 14% of the blasts) and impaired MTX transport (in 17% of the blasts). Our previous study (Matherly et al, 1995) showed that MTX transport was functionally intact in previously cryopreserved ALL specimens.

### Confocal microscopy experiments

Log-phase K562, K500E and K43-6 cells were incubated at 37°C with 1 μM F-MTX in culture medium supplemented with 16 μM thymidine and 100 μM hypoxanthine for up to 24 h. Control incubations were performed in the presence of either 500 μM MTX or 100 μM ZD1694. After incubations of 3, 5, 15, 60 min and 6 and 24 h, the cells were fixed with a freshly prepared solution of 4% paraformaldehyde in phosphate-buffered saline (PBS) for 20 min at 4°C and rinsed three times with ice-cold PBS. The cells were mounted on slides with Aquapolymount for confocal microscopic analysis. For the experiments with the patient blasts, cells were incubated in Hanks' balanced salts with 1 μM F-MTX with and without 100 μM ZD1694 at 37°C for 15, 30 and 60 min. Cell densities were 1.5–3 million cells per 600 μL. The cells were fixed, washed and mounted as described above.

Confocal microscopic images were acquired on a Leica confocal scanning laser microscope. Scanning was made at 4.0 electronic zoom so that at a magnification of × 40 (using a fluotar oil immersion objective) the final $(x,y)$ resolution was 1 pixel per 0.245 μm. Images of single cells were acquired by putting one optical section through the cell centre with 32 scans per frame. For all acquisitions, the gain and black levels were set manually to optimize the dynamic range of the image while ensuring that no region was completely suppressed (intensity = 0) or completely saturated (intensity = 255). All images were stored on an optical disk and printed using a Focus camera system that used false colours to simulate fluorescence intensity. Representative pictures of the fluorescent patterns observed in the different experiments performed are shown in Figures 1 and 2. Each experimental condition illustrated for the cell line experiments (Figure 1) was carried out on three different occasions. The patient experiments (Figure 2) were only perfomed twice because of the limited availability of samples. A total of 100 cells per slide was analysed and scanned. The selected field for analysis was chosen randomly on each slide. There was a very good homogeneity in the fluorescent patterns observed among different cells on the same slides. In the experimental conditions in which cells were labelled, about 70–75% of the cells were fluorescent with consistent intensity and labelling pattern.

### RESULTS

Confocal microscopy images for wild-type K562 cells (A–D), their transport deficient subline (K500E; E–H) and K500E cells transfected with the human RFC cDNA (designated K43-6; I–L) are shown in Figure 1, following incubations with 1 μM F-MTX. In the wild-type K562 line, intracellular fluorescence labelling was maximal as early as 5 min after exposure to F-MTX (Figure 1A shows the labelling at 15 min) and was highly homogeneous; most of the fluorescence was localized in the cytosol. In the presence of 100 μM ZD1694 (Figure 1B) or 500 μM MTX (not shown), F-MTX fluorescence uptake was virtually completely abolished during incubations up to 60 min, establishing a role for the RFC in F-MTX uptake over this interval. Although there was a complete absence of F-MTX uptake over 60 min in K500E cells (Figures 1E and F), fluorescence uptake was re-established in the K43-6 transfectants (Figure 1I). The fluorescence uptake in K43-6 cells was more heterogeneous (not shown) and was frequently more intense than for the wild type cells. Further, F-MTX uptake could be blocked with ZD1694 (Figure 1J).

Although the early time course labelling data strongly suggested a RFC-mediated uptake component for F-MTX, cellular fluorescence accumulations were essentially identical in all cell lines following sustained F-MTX exposures (24 h; Figures 1C, G and K). Furthermore, co-incubation with ZD1694 did not affect F-MTX labelling at 24 h (Figures 1D, H and L). In contrast, MTX (500 μM) partially decreased fluorescence in wild-type cells at this time (not shown), presumably because of competition for DHFR binding.

Confocal images of F-MTX uptake by the leukaemic blast cells from children with ALL are illustrated in Figure 2. Blast cells were significantly smaller than the cultured cell lines and typically exhibited large nuclei and greatly reduced cytosol. Two of the blast specimens (no. 2 and no. 26) previously reported to have intact RFC transport (Matherly et al, 1995), accumulated high levels of F-MTX over 15 min that appeared as intense, perinuclear punctate fluorescence (Figure 2A and B respectively). As for the cultured cells, cellular fluorescence was maximal within 5 min and was essentially unchanged for up to 60 min and was nearly completely abolished in

© Cancer Research Campaign 1997

the presence of 100 μM ZD1694 (not shown). For a B-precursor specimen (no. 12) with a documented impairment in MTX uptake (Matherly et al, 1995), there was no detectable F-MTX accumulation in over 90% of the blasts examined (Figure 2C).

### DISCUSSION

The enhanced image resolution of confocal microscopic analysis and the possibility of doing a series of optical sections of the human leukaemic cells studied, allowed for the intracellular localization of the F-MTX probe. (Although F-MTX is primarily localized to the cytosol rather than sequestered in organelles such as the mitochondria and lysosomes, its distribution to these compartments cannot be discounted. Further, it is not inconceivable that because of its lack of polyglutamylation, F-MTX may exhibit a different intracellular distribution from methotrexate.) It also allowed us to observe a RFC-specific component of F-MTX uptake that could not be identified by conventional fluorescence microscopy or flow cytometry. Fluorescence was barely visible by conventional microscopy in the leukaemic cell lines and we could not reproducibly document F-MTX uptake by flow cytometry after short F-MTX incubations. During short time exposures to F-MTX (< 15 min), no appreciable increase in relative fluorescence was detected over high background autofluorescence. Only after prolonged drug exposures (> 6 h) was there a sufficient fluorescent signal for dependable flow cytometry detection (data not shown). The recent cloning of RFC cDNAs (Dixon et al, 1994; Williams et al, 1994; Moscow et al, 1995; Williams and Flintoff, 1995; Wong et al, 1995) and the availability of a RFC-deficient human cell line stably transfected with a RFC cDNA (Wong et al, 1997) allowed us to directly establish that F-MTX uptake *during short incubations* was mediated by the RFC. Although F-MTX labelling was highly homogeneous in wild type K562 and K500E sublines, in the RFC transfectants fluorescence was more heterogeneous and, frequently, more intense than wild-type cells. The latter finding is consistent with greater RFC expression (15-fold) and transport (twofold) in transfected over wild-type cells (Wong et al, 1997). Morever, both the K500E and K43-6 lines exhibit a 7.7-fold increased DHFR content over wild-type cells (Wong et al, 1997).

The RFC-specific uptake of F-MTX was further confirmed by co-incubation studies with MTX and ZD1694. Both these antifolates preferentially use the RFC for transmembrane transport (Jackman et al, 1991; Westerhof et al, 1995) and almost completely prevented F-MTX uptake in RFC-competent cells during short incubations. The decrease in cellular labelling by the thymidylate synthase inhibitor ZD1694 confirmed that this observation could not be simply explained by intracellular probe displacement from DHFR. F-MTX did accumulate in both RFC-competent and deficient cells after longer incubations, presumably via nonspecific diffusion (Henderson et al, 1980; Assaraf et al, 1989). [Although the nature of this process is unestablished, it is clear that it does not involve RFC. Based on folic acid growth requirements and our inability to detect immunoreactive folate receptors on Western blots of membrane proteins probed with antiserum to human folate receptor (unpublished data), it appears unlikely that high-affinity membrane folate receptors (Antony, 1992) play any significant role in the uptake of F-MTX in the K562 sublines.] Under these conditions, partial probe displacement from dihydrofolate reductase was seen during MTX co-incubation but not with ZD1694; the former probably reflects partial probe displacement from dihydrofolate reductase by MTX, as described previously (Assaraf and Schimke, 1987). Notably, displacement of F-MTX cell labelling by MTX could not be identified in the RFC-transfectants under the same conditions (data not shown). However, this may simply reflect the greater labelling of these cells compared with the wild-type cells, as noted above.

Therefore, our report is the first demonstration of a RFC-mediated component for F-MTX cellular uptake in tumour cells. F-MTX binding to RFC was previously documented in L1210 cells, but cellular F-MTX uptake, monitored by fluorimetry, was very slow and largely independent of the RFC (Henderson et al, 1980; Fan et al, 1991). Although no specific interaction between F-MTX and the RFC could be documented in Chinese hamster ovary cells (Assaraf et al, 1989), this may be due to the low sensitivity of the flow cytometry method used (see above). This observation may also reflect differences in anti-folate affinities between human and hamster RFC proteins (Wong et al, 1995). Of course, as noted above, in all the K562 sublines, F-MTX uptake during prolonged exposures was also independent of RFC.

Qualitatively identical results were obtained for F-MTX uptake into leukaemic blasts from children with ALL. Therefore, for two specimens previously reported with intact RFC transport, F-MTX accumulated to high levels and was nearly completely abolished by co-incubation with ZD1694. The labelling intensity in these samples was more intense than either the wild type or transfected K562 cells; this probably reflects the significantly reduced cytoplasmic content of leukaemic blasts. It was of particular interest that an additional ALL specimen (sample 12), with impaired MTX transport by flow cytometry (Matherly et al, 1995), similarly exhibited impaired F-MTX uptake by confocal microscopy. Although only 14% of the blasts were previously reported with defective MTX transport, when assayed with F-MTX and confocal microscopy, F-MTX uptake was completely suppressed in over 90% of the cells. This seeming discrepancy probably reflects differences in sensitivity between our earlier indirect flow cytometry assay of RFC transport in ALL specimens (Matherly et al, 1995) and direct visualization of transport by confocal microscopy. High concentrations of displacing MTX may preclude detection of low levels of MTX transport impairment by flow cytometry and low levels of dihydrofolate reductase interfere with the detection of incomplete F-MTX displacement in transport-defective cells. Our results suggest the potential of confocal analysis as a tool for visualizing cellular events involved in anti-folate uptake and accumulation, including clinical resistance. It will be essential to apply these methods to a large patient population to establish their general use.

### ACKNOWLEDGEMENTS

We thank Dr F. M. Huennekens for his generous advice and Ms Rachenii Ekizian for assistance with the ALL experiments. This work was supported in part by United States PHS grant CA-53535, American Cancer Society Grant EDT-105, a grant from the United Way of Michigan, and a Scholar Award from the Leukaemia Society of America, Inc.

### REFERENCES

Antony AC (1992) The biological chemistry of folate receptors. *Blood* **79**: 2807–2820

Assaraf YG and Schimke RT (1987) Identification of methotrexate transport deficiency in mammalian cells using fluoresceinated methotrexate and flow cytometry. *Proc Natl Acad Sci USA* **84**: 7154–8

© Cancer Research Campaign 1997

Assaraf Y, Seamer L and Schimke R (1989) Characterization by flow cytometry of fluorescein-methotrexate transport in Chinese hamster ovary cells. *Cytometry* **10**: 50–55

Barredo JC, Synold TW, Laver J, Relling MV, Pui CH, Priest DG and Evans WE (1994) Differences in constitutive and post-methotrexate folylpolyglutamate synthetase activity in B-lineage and T-lineage leukemia. *Blood* **84**: 564–569

Buonocore L and Rose JK (1991) Liposome-mediated transfection. In *Current Protocols in Molecular Biology*, Asubel FA, Brent R, Kingston RE, Moore DD, Seidman JG, Smith JA and Struhl K (eds), pp. 9.4.2–9.4.4

Dixon KH, Lanpher BC, Chiu J, Kelley K and Cowan KH (1994) A novel cDNA restores reduced folate carrier activity and methotrexate sensitivity to transport deficient cells. *J Biol Chem* **269**: 17–20

Fan J, Pope LE, Vitols KS and Huennekens, FM (1991) Affinity labelling of folate transport proteins with the N-hydroxysuccinimide ester of the gamma-isomer of fluorescein-methotrexate. *Biochemistry* **30**: 4573–4580

Goldman ID and Matherly LH (1985) The cellular pharmacology of methotrexate. *Pharmacol Ther* **28**: 77–102

Henderson GB, Russell A and Whiteley JM (1980) A fluorescent derivative of methotrexate as an intracellular marker for dihydrofolate reductase in L1210 cells. *Arch Biochem Biophys* **202**: 29–34

Jackman AL, Taylor GA, Gibson W, Kimbell R, Brown M, Calvert AH, Judson IR and Hughes LR (1991) ICI–D1694, a quinazoline antifolate thymidylate synthase inhibitor that is a potent inhibitor of L1210 tumor cell growth in vitro and in vivo. *Cancer Res* **51**: 5579–5586

Jolivet, J, Cowan KH, Curt GA, Clendeninn NJ and Chabner BA (1983) The pharmacology and clinical use of methotrexate. *N Engl J Med* **309**: 1094–1104

Kaufman RJ, Bertino JR and Schimke RT (1978) Quantitation of dihydrofolate reductase in individual parental and methotrexate-resistant murine cells. Use of a fluorescence activated cell sorter. *J Biol Chem* **253**: 5852–5860

Matherly LH, Angeles SM and Czajkowski CA (1992) Characterization of transport-mediated methotrexate resistance in human tumor cells with antibodies to the membrane carrier for methotrexate and tetrahydrofolate cofactors. *J Biol Chem* **267**: 23253–23260

Matherly LH, Taub JW, Ravindranath Y, Proefke SA, Wong SC, Gimotty P, Buck S, Wright JE and Rosowsky A (1995) Elevated dihydrofolate reductase and impaired methotrexate transport as elements in methotrexate resistance in childhood acute lymphoblastic leukaemia. *Blood* **85**: 500–509

Moscow JA, Gong MK, He R, Sgagias MK, Dixon KH, Anzick SL, Meltzer PS and Cowan KH (1995) Isolation of a gene encoding a human reduced folate carrier (Rfc1) and analysis of its expression in transport-deficient, methotrexate-resistant human breast cancer cells. *Cancer Research* **55**: 3790–3794

Pizzo PA and Poplack DG (1993) *Principles and Practice of Pediatric Oncology*. Lippincott: Philadelphia, PA

Rosowsky A, Wright JE, Shapiro H, Beardsley P and Lazarus H (1982) A new fluorescent dihydrofolate reductase probe for studies of methotrexate resistance. *J Biol Chem* **257**: 14162–14167

Synold TW, Relling MV, Boyett JM, Rivera GK, Sandlund JT, Mahmoud H, Crist WM, Pui CH and Evans WE (1994) Blast cell methotrexate–polyglutamate accumulation in vivo differs by lineage, ploidy, and methotrexate dose in acute lymphoblastic leukaemia. *J Clin Invest* **94**: 1996–2001

Trippett T, Schlemmer S, Elisseyeff Y, Goker E, Wachter M, Steinherz P, Tan C, Berman E, Wright JE, Rosowsky A, Schweitzer B and Bertino JR (1992) Defective transport as a mechanism of acquired resistance to methotrexate in patients with acute lymphocytic leukaemia. *Blood* **80**: 1158–1162

Westerhof GR, Rijnboutt S, Schornagel JH, Pinedo HM, Peters GJ and Jansen G (1995) Functional activity of the reduced folate carrier In Kb, Ma104, and Igrov-I cells expressing folate-binding protein. *Cancer Res* **55**: 3795–3802

Whitehead VM, Rosenblatt DS, Vuchich MJ, Shuster JJ, Witte A and Beaulieu D (1990) Accumulation of methotrexate and methotrexate polyglutamates in lymphoblasts at diagnosis of childhood acute lymphoblastic leukemia: a pilot prognostic factor analysis. *Blood* **76**: 44–49

Whitehead VM, Vuchich MJ, Lauer SJ, Mahoney D, Carroll AJ, Shuster JJ, Esseltine DW, Payment C, Look AT, Akabutu J, Bowten T, Taylor LD, Camitta B and Pullen DJ (1992) Accumulation of high levels of methotrexate polyglutamates in lymphoblasts from children with hyperdiploid (greater than 50 chromosomes) B-lineage acute lymphoblastic leukemia: a Pediatric Oncology Group study. *Blood* **80**: 1316–1323

Williams FM and Flintoff WF (1995) Isolation of a human cDNA that complements a mutant hamster cell defective in methotrexate uptake. *J Biol Chem* **270**: 2987–2992

Williams FM, Murray RC, Underhill TM and Flintoff WF (1994) Isolation of a hamster cDNA clone coding for a function involved in methotrexate uptake. *J Biol Chem* **269**: 5810–5816

Wong SC, Proefke SA, Bhushan A and Matherly LH (1995) Isolation of human cDNAs that restore methotrexate sensitivity and reduced folate carrier activity in methotrexate transport-defective Chinese hamster ovary cells. *J Biol Chem* **270**: 17468–17475.

Wong SC, McQuade R, Proefke SA, Bhushan A and Matherly LH (1997) Human K562 transfectants expressing high levels of reduced folate carrier but exhibiting low transport activity. *Biochem Pharmacol* **53**: 199–206

© Cancer Research Campaign 1997